# Laypersons can successfully place supraglottic airways with 3 minutes of training. A comparison of four different devices in the manikin

**DOI:** 10.1186/1757-7241-19-60

**Published:** 2011-10-24

**Authors:** Gereon Schälte, Christian Stoppe, Meral Aktas, Mark Coburn, Steffen Rex, Marlon Schwarz, Rolf Rossaint, Norbert Zoremba

**Affiliations:** 1 Department of Anesthesiology, University Hospital Aachen, Aachen, Germany; 2 Department of Pediatrics and Neonatology, University Hospital Aachen, Aachen, Germany

## Abstract

**Introduction:**

Supraglottic airway devices have frequently been shown to facilitate airway management and are implemented in the ILCOR resuscitation algorithm. Limited data exists concerning laypersons without any medical or paramedical background. We hypothesized that even laymen would be able to operate supraglottic airway devices after a brief training session.

**Methods:**

Four different supraglottic airway devices: Laryngeal Mask Classic (LMA), Laryngeal Tube (LT), Intubating Laryngeal Mask (FT) and CobraPLA (Cobra) were tested in 141 volunteers recruited in a technical university cafeteria and in a shopping mall. All volunteers received a brief standardized training session. Primary endpoint was the time required to definitive insertion. In a short questionnaire applicants were asked to assess the devices and to answer some general questions about BLS.

**Results:**

The longest time to insertion was observed for Cobra (31.9 ± 27.9 s, range: 9-120, p < 0.0001; all means ± standard deviation). There was no significant difference between the insertion times of the other three devices. Fewest insertion attempts were needed for the FT (1.07 ± 0.26), followed by the LMA (1.23 ± 0.52, p > 0.05), the LT (1.36 ± 0.61, p < 0.05) and the Cobra (1.45 ± 0.7, p < 0.0001). Ventilation was achieved on the first attempt significantly more often with the FT (p < 0.001) compared to the other devices. Nearly 90% of the participants were in favor of implementing supraglottic airway devices in first aid algorithms and classes.

**Conclusion:**

Laypersons are able to operate supraglottic airway devices in manikin with minimal instruction. Ventilation was achieved with all devices tested after a reasonable time and with a high success rate of > 95%. The use of supraglottic airway devices in first aid and BLS algorithms should be considered.

## Introduction

The securing of the airway and ventilation of the lungs is of paramount importance following initial chest compressions during cardiopulmonary resuscitation (CPR). In the preclinical setting, physical contact with the patient (in particular their mouth) presents a strong deterrent to many lay responders. Disgust and fear of infection, associated with contact with bodily fluids are frequently cited as preventing immediate care [1-3]. This may coincide with a fear of incorrect mouth-to-mouth ventilation and potential malpractice consequences.

To facilitate mouth-to-mouth ventilation in case of out-of-hospital CPR several products are available. These products are based on the principle of either covering the mouth and/or face with a drape, or covering the nose and mouth via facemask with a mouth adapter. All devices are equipped with a protective filter system. Achieving adequate ventilation (without gastric inflation) is dependent on the seal of either face mask or drape, manual skills, and the acceptance of close physical contact with a stranger and the associated risks [3]. Independent of the type of device, it must be readily available, e.g. close to automatic external defibrillators (AEDs), or carried in the pocket in readiness for emergencies.

For both junior and experienced medical personnel, supraglottic airway devices have frequently been shown to facilitate airway management. Therefore, the laryngeal mask and laryngeal tube are implemented in the American Society of Anesthesiologists (ASA) algorithm for the management of the difficult airway and the International Liaison Committee on Resuscitation (ILCOR) algorithm for cardiopulmonary resuscitation [4]. In both, manikin and clinical studies, Paramedics, nurse and (para-) medical students have been shown to secure the airway and ventilate the lungs faster and more effectively when using a supraglottic airway device compared to mouth-to-mouth ventilation [5-8]. In a recently published study we demonstrated that even without any instruction, first year medical students were able to insert a supraglottic airway device intuitively with a reasonable speed and success rate. After a minimal well directed training, insertion times and success rates can be markedly improved [9].

We therefore hypothesized that even laypersons without any medical background would be able to operate supraglottic airway devices following brief instruction.

The aims of this study were: 1) to test whether lay persons are able to secure the airway and ventilate the lungs adequately using a supraglottic device following a brief training session of 3 minutes; and 2) to compare four supraglottic devices with regard to practicability: Laryngeal Mask Classic^® ^(LMA) and Laryngeal Mask Fastrach^® ^(FT) (both: LMA Deutschland GmbH, Bonn, Germany), Laryngeal Tube^® ^(LT) (VBM Medizintechnik GmbH, Sulz, Germany), CobraPLA^® ^(Cobra) (Engineered Medical Systems, Indianapolis, IL, USA).

## Methods

The institutional review board waived the requirement to obtain written informed consent from the participants as no personal data except age and first aid knowledge were collected, and no influence on the participants' health was expected. All subjects agreed for their performance to be evaluated and anonymously used for scientific and educational purposes. Prerequisites for inclusion were the lack of any previous medical education (i.e. physician, nurse, EMT, paramedic) other than a "first-aid" course, and an age of 16 or older.

Applicants were recruited in a public shopping mall and in the central cafeteria of the RWTH Aachen University campus. Experimental data were recorded "on-site".

Four different supraglottic airway devices (LMA, LT, FT and Cobra) were investigated. The order in which devices were presented was rotated after every 35 participants to eliminate any bias. A resuscitation scenario with a manikin (Ambu Airway Man^®^, Ambu GmbH, Bad Nauheim, Germany) laying on the floor was prepared. All participants received a single minimal standardized training session "hands off" before their individual trial. The instructions comprised the following sentences: "*This patient is in respiratory arrest. He has stopped breathing and you must begin ventilation immediately. Much better and more efficient than mouth-to-mouth ventilation is the use of one of these 4 devices (demonstrated). Simply take one of them, insert it into the mouth of the patient, with the opening facing in the same direction as the "belly button" until you feel resistance, connect the syringe, inflate the balloon to form a seal (demonstrated with the LMA), connect the bag-valve and start to ventilate the manikins lungs by compressing the bag gentle but full squeeze (two hands, demonstrated). If the thorax does not expand and this indicator (shown) does not show green or yellow, immediately take the device out, deflate the balloon, and start again (demonstrated)"*. The procedure was thus demonstrated step by step during the verbal instructions. We chose the LMA for demonstration representing the eldest and most reviewed modern supraglottic airway device. At the end the procedure was demonstrated again from start to finish. Thereafter, no further questions were answered, nor was the demonstration repeated on request. Immediately following insertion of the device a prepared syringe with the designated correct air volume was connected, the cuff was inflated, and the time to first manual ventilation was recorded. The volume of air inflated (Ambu Airway Man^® ^scale) was measured and any eventual leak estimated. The cuffs of all supraglottic devices were inflated with the maximum volumes of air recommended by the manufacturer (LMA size 4 and FT size 4 with 30 ml each, Cobra size 4 with 70 ml, and LT size 4 with 80 ml). A single trial was aborted after 2 minutes or more than 3 failed attempts.

A tidal volume of > 500 ml was considered as sufficient according to the ERC resuscitation guidelines. A tidal volume of less than < 500 ml was deemed insufficient. An expiratory tidal volume > 800 ml was classed as no leakage, 500-799 ml as minor leakage and < 500 ml as major leakage.

To eliminate the bias of a potential learning curve by the sequence supraglottic devices were changed in random-order. None of the participants could watch the trial of any other participator. Partition panels were used to separate and hide the resuscitation scenario.

At the end, individuals were asked which of the 4 devices they preferred. Finally 5 questions concerning resuscitation had to be answered yes or no.

1. If emergency resuscitation kits including a bag-valve and a supraglottic airway device were available in public places, e.g. sports facilities or at your workplace, do you believe you would be able to use them to deliver adequate lung ventilation?

2. Do you believe that the combination of a supraglottic airway device and a bag-valve can be used by lay responders during resuscitation reasonably?

3. Should this kind of lung ventilation be taught in first aid classes?

4. Do you think that this type of lung ventilation would make you more willing to deliver lung ventilation during cardiopulmonary resuscitation?

### Statistics

A success rate of 95% was expected [10,11]. The power of the study was calculated with a significance level, α = 0.05. The equivalence limit difference, d0 was assumed to be 7 and the expected difference, d1 was set to be 0. A power of 80% results in a sample size of 120. In total 141 study subjects were included to compensate for possible dropouts. The power calculation was performed using nQuery Advisor^® ^Version 7.0 (Statistical Solutions, Saugus, MA, USA).

Statistical analysis was performed using GraphPad Prism 5.0 for Mac (GraphPad Software, San Diego, CA, USA). Metric scaled data were analyzed calculating mean and standard deviation. Analysis of variance (ANOVA) with Bonferoni correction for multiple comparisons was used to detect statistical differences between the groups. To analyze the variation in repeated measures of tidal volume, number of attempts, and time to insertion, Friedmann's test with Dunn's correction was used. The level of significance was set at 5% for these four variables. A P of ¼*5% = 0.0125 indicated statistical significance. A chi-square test was used to detect statistical differences in contingent data.

## Results

Data from 141 subjects (65 women, 76 men) were analyzed. None of these had any previous medical or paramedical education. In addition 19.1% (n = 27) of these individuals had never participated in a first-aid course, whereas a first aid course had been taken more than 10 years ago in 13.5% (n = 19), 2-10 years ago in 43.3% (n = 61) and less than 2 years ago in 24.1% (n = 34) of the participants (Table 1).

**Table 1:** Demographical data and previous first aid knowledge

Characteristics	
Age, y	24.96 ± 8 [16-73]
Sex	
female, n	65
male, n	76
First aid education, n	
no	27 (19.1%)
< 2 years	34 (13.5%)
2-10 years	61 (43.3)
> 10 years	19 (13.5%)

Data are presented as mean +/- standard deviation, [range], numbers and (percentage)

**Figure 1:**
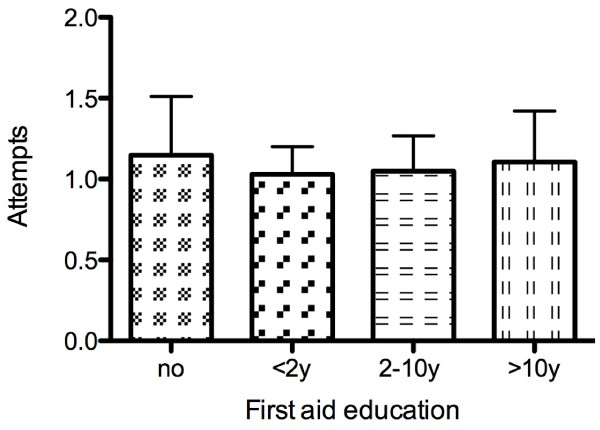
**Time for insertion dependent on previous first aid education**. No correlation was found between the time of insertion and the time passed since or no "first aid" education (p = 0.29). Data are presented as mean ± SD.

**Figure 2:**
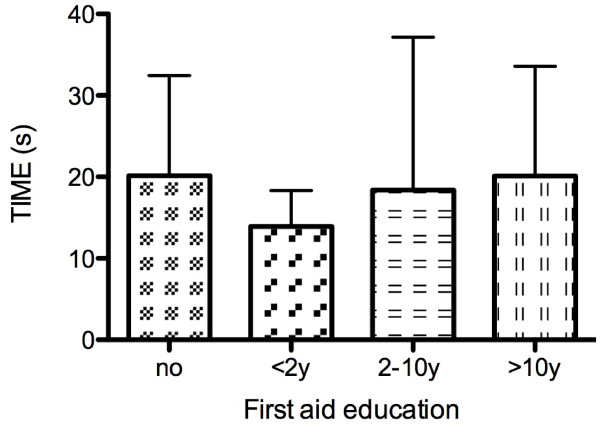
**Number of attempts needed dependent on previous first aid education**. No correlation was found between the number of attempts required and the time passed since or no "first aid" education (p = 0.25). Data are presented as mean ± SD.

Neither first-aid training itself nor how current this was correlated either with the time to insertion (p = 0.29) (Figure 1) or the number of attempts required (p = 0.25) (Figure 2).

**Figure 3:**
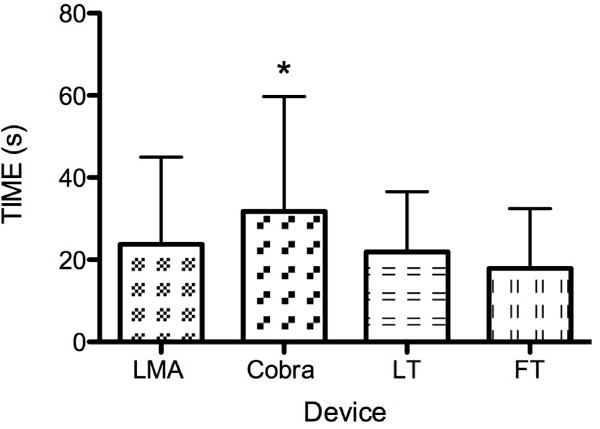
**Time of insertion dependent on the supraglottic device**. Cobra needed the longest time for insertion (p < 0.0001)*. No statistically significant differences between the insertion times of the other three devices were found. Data are presented as mean ± SD.

A comparison of the insertion times between the LMA, the LT, the FT and the Cobra devices showed the Cobra to need the longest time for insertion (p < 0.0001). No statistically significant differences between the insertion times of the other three devices were found Figure 3).

**Figure 4:**
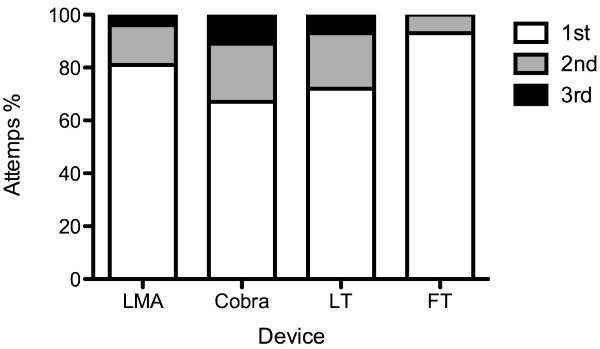
**Number of attempts needed dependent on the supraglottic device**. FT required the fewest attempts, followed by the LMA, the LT and the Cobra. No statistically significant differences could be shown between the FT and the LMA (p > 0.05). Compared to the FT, the Cobra* (p < 0.0001) and LT^# ^(p < 0.05) needed significantly more attempts for correct placement. No significant differences between the Cobra, LMA, and LT were found. Data are presented in percentage.

**Figure 5:**
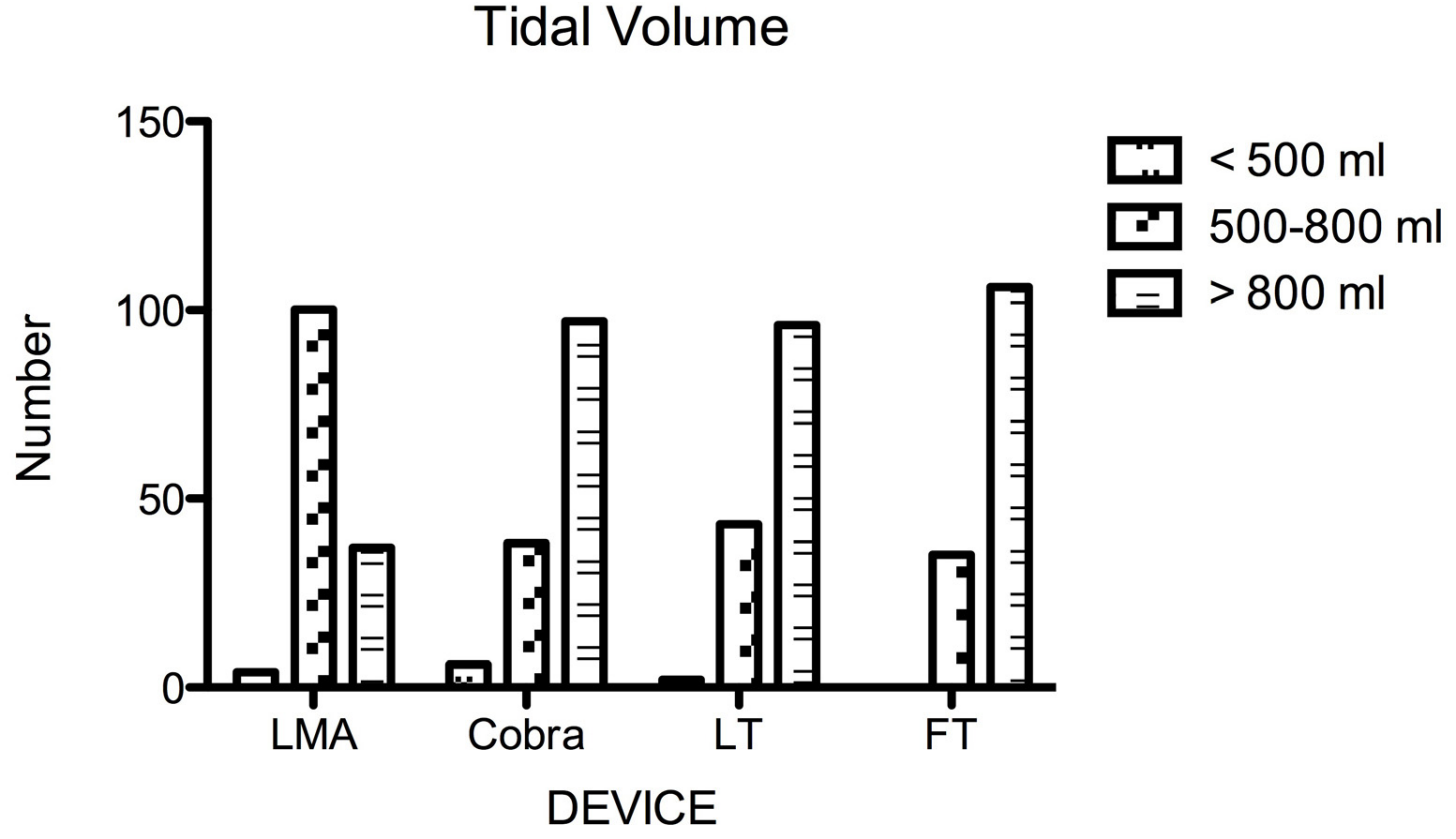
**Applied tidal volumes dependent on the supraglottic device**. No significant differences in the application of "sufficient" tidal volumes (> 500 ml) were found among the 4 devices (p = 0.08). Analysis of subgroups with tidal volumes of 500-800 ml and > 800 ml found significantly smaller but sufficient tidal volumes (500-800 ml) in LMA* ventilated patients (p < 0.0001). Data are presented in numbers.

Regarding ease of insertion, the FT (1.07 ± 0.26) required fewest attempts, followed by the LMA (1.23 ± 0.52), the LT (1.36 ± 0.61) and the Cobra (1.45 ± 0.7). No statistically significant differences could be shown between the FT and the LMA (p > 0.05). Compared to the FT the Cobra (p < 0.0001) and LT (p < 0.05) required significantly more attempts for correct placement, but no significant differences between the Cobra, LMA, and LT were found. Significantly more patients were ventilated on the first attempt with the FT (p < 0.001) (Table 2). No significant differences in the application of "sufficient" tidal volumes (> 500 ml) were found among the 4 devices (p = 0.08). Analysis of subgroups with tidal volumes of 500-800 ml and > 800 ml found significantly smaller (but sufficient) tidal volumes (500-800 ml) in LMA ventilated patients (p < 0.0001) (Figure 4). With all tested supraglottic devices a tidal volume of more than 150 ml-the estimated dead space-could be generated (Figure 5).

**Table 2:** Time of insertion, attemps and ventilation quality (tidal volumes, estimated leakage).

	LMA	Cobra	LT	FT
Time to insertion	23.8 ± 21.2 (17) [9-120]	31.9 ± 27.9 (19) [9-120]	21.9 ± 14.7 (15) [8-75]	17.9 ± 14.6 (15) [8-120]
Success, n	138 (97.1%)	105 (95.8%)	139 (98.6%)	141 (100%)
Leakage, tidal volumes				
no, > 800 ml	36 (25.5%)	96 (68.1%)	97 (68.8%)	107 (75.9%)
minor, 500-800 ml	101(71.6%)	39 (27.7%)	42 (29.8%)	34 (24.1%)
major, < 500 ml	4 (2.8%)	6 (4.3%)	2 (1.4%)	0
Attempts,%				
1^st^/2^nd^/3^rd^	80.9/14.9/4.3	66.7/22/11.3	71.6/21.3/7.1	92.9/7.1/0

Tidal volumes > 500 ml were quoted sufficient according the ILCOR guidelines. With all devices a tidal volume greater than 150 ml (estimated dead space) could be applied. Data are presented as mean +/- standard deviation, (median), [range] and number.

In their statements evaluating the 4 devices subjects expressed a preference for the FT (41.8%, n = 59), followed by the LMA (34%, n = 48), the LT (20.6%, n = 29) and the Cobra (3.5%, n = 5). Commonly cited as supporting their classification were the ease of handling, how intuitive they were to use, and their "grip".

Failed insertions due to incorrectly rotated devices occurred 15 times. All of these occurred in Cobra and on the 1^st ^attempt (6× upside-down, p = 0.67; 9× 180° dorso-ventral rotation & 1 combined upside-down, p = 0.0024).

No bias of a potential "learning curve" by sequence of the supraglottic devices could be demonstrated in terms time of insertion and number of attempts (Table 3).

**Table 3:** Influence of sequence on insertion time and number of attemps

Sequence	1	2	3	4	P value
Time [s]					
LMA	28 ± 21.4	19.7 ± 15.8	25.3 ± 25.9	22 ± 21	0.36
Cobra	26.7 ± 18.4	34.8 ± 35.1	29.6 ± 25.3	35.9 ± 30.5	0.47
LT	19.6 ± 13.4	20.74 ± 19.9	23.4 ± 13.9	23.8 ± 17.4	0.57
FT	22.5 ± 25.1	15.7 ± 5.9	14.9 ± 5.7	18.4 ± 11.7	0.13
Attempts [n]					
LMA	1.36 ± 0.59	1.14 ± 0.35	1.22 ± 0.54	1.12 ± 0.53	0.31
Cobra	1.37 ± 0.59	1.45 ± 0.78	1.37 ± 0.59	1.58 ± 0.77	0.52
LT	1.25 ± 0.56	1.32 ± 0.58	1.36 ± 0.59	1.48 ± 0.7	0.46
FT	1.02 ± 0.35	1.02 ± 0.16	1.02 ± 0.16	1.08 ± 0.28	0.19

No significant difference between LMA, Cobra, LT and FT regarding the sequence of use could be demonstrated for the time of insertion or the attemps needed. Data are presented as means +/- standard deviation

Questionnaire: 67.4% of the participants felt competent in performing ventilation with a supraglottic airway after this brief training, and 87.9% judged the combination of a supraglottic airway and a bag-valve to be a useful aid in out-of-hospital resuscitation. Nearly 89.4% supported the introduction of and briefing with supraglottic airway devices in first-aid courses. Finally, 85.8% agreed that the availability of these devices would make them more likely to attempt ventilation during cardiopulmonary resuscitation.

## Discussion

In this manikin study, we show that laypersons are able to successfully place a supraglottic airway following minimal training.

This ability was independent of prior first-aid education or its age. Despite the inherent limitations of the scenario (plastic device inserted into plastic mannequin) and the limited malleability of the "oral and supraglottic tissue" of the Ambu Airway Man^®^, we show that all four supraglottic airway devices applied by laymen provided a reasonable airway and allowed for the application of sufficient tidal volumes.

FT performed best with regard to time to insertion, number of attempts required, and in the subjective assessments of the participants. Interestingly, none of the four devices tested actually "failed". A tidal volume of more than the assumed dead space (< 150 ml) could always be generated.

In contrast to previous studies, none of our volunteers had any healthcare training other than first aid provider courses. We consider them therefore to act more intuitively and with a lower level of caution than healthcare professionals, who are more familiar with and likely more fearful of potential adverse effects.

Previous studies in patients, cadavers and manikin have all proven LMA, FT, LT and Cobra to be efficient tools for airway management in the hands of naïve "intubators" and inexperienced medical personnel [5,6,12-16]. These studies investigated first year medical, paramedical and nursing students, as well as military personnel in combat paramedical or medical education. In all these subjects we might assume a certain interest in, familiarity with and aptitude for a range of medical procedures and emergencies. In addition, a current certification in first-aid is a prerequisite for all such vocations (whereas it is not required to hold a driving license, for example). Therefore, although not (yet) formally trained in healthcare we might expect such subject populations to perform differently to "true laypersons" as per our definition.

In the present study more than 58% of the participants had not attended a first aid course in the past 2 years and 19% had no first aid training at all. (Only 23% of subjects had attended a first-aid course within the past 2 years). Beauchamp et al. as well found a high success rate independent from previous "first-aid" education [17].

To the best of our knowledge, this is the first study comparing several supraglottic airway devices focusing on "true laypersons" without any background in healthcare, and in some cases without basic first-aid training.

The participants in previous studies, in addition to a more healthcare oriented background, also received a longer and more comprehensive training session prior to intonation. Few studies have focused on the use of supraglottic airway devices by subjects having received minimal training prior to intubation [9,18,19]. Jokela et al. used a short educational video-clip for instruction and demonstrated that inexperienced first responder trainees could secure the airway in a manikin with the FT and the LT with a comparable success rate [10]. In contrast, we found a significantly better performance of laypersons operating the FT. A similar result has previously been reported for paramedical students [20], non-anesthetic medical staff and non-medical staff [9,12,19].

Also in accordance with our results, the success rate for intubation attempts and the insertion times for the COBRA device have previously been demonstrated to be lower than for other devices, as well as being critically dependent upon operator experience [15].

The present study also observed another important problem with the COBRA-device: in 9 cases it was inserted 180° rotated, with the airway aperture dorsal instead of ventral. In the Cobra, the airway aperture may be overseen due to the lack of curvature in the stem and the fact that the aperture itself appears covered by some bars. Furthermore, the blocking balloon encircles the distal part of the device. Consequently, the COBRA device performed worse than the LM even when inserted by experienced anesthetists [13]. Although in our study an attempted upside-down insertion of the COBRA-device was immediately recognized and corrected by the participants, it remains questionable whether in the setting of a real resuscitation lay responders under stress would act similarly. The difficulties described above likely contribute to the participants estimation of the COBRA as the most difficult and inconvenient device. The LT and FT were judged as most intuitive.

Success rates for the LT as observed in our study are similar to those reported in the literature. Although Wrobel et al. indicated that lack of clinical experience could halve the initial success rate when the LT is inserted by non-anesthesiologists, our results do not reveal such a low success rate for the LT [21]. Similarly, in an out-of-hospital trial investigating LT airway management by paramedics and emergency physicians, both performed equally well when using the LT as a rescue device after failed endotracheal intubation or as an initial airway [22]. The majority of users were relatively inexperienced, with less than 5 LT placements. In accordance with our data first attempt success was about 78%. Moreover, in undergraduate students without medical training a high success rate of 80% for the King LT-D could be demonstrated using "on-site" minimal scripted telephonic instruction [17].

In a manikin untrained laypersons can achieve a secured airway with the LMA or LMA-classic even without detailed background knowledge about the tool. In fact, minimal theoretical instruction and practical skill training significantly improved their performance [9]. LMA supreme, in novice hands, systematically promoted easier ventilation of better quality than the facemask in morbidly obese patients showing difficult mask ventilation predictors [23]. The authors suggest that the LMA Supreme could be considered as a standard airway management tool for both elective and rescue airway management. A 100% success rate in manikin and a 64% success rate in the field among adult out-of-hospital non-traumatic cardiac arrest have been reported in paramedics with manikin training only [20]. Timmermann et al. demonstrated that medical students could establish ventilation with the intubation laryngeal mask significantly more successful and rapidly compared to bag-valve ventilation in anesthetized patients [24]. In a single trial "mouth-to-mouth ventilation" via a pocket face-mask was compared to the LMA provided by basic trained nurses. Success rate were 51% for "mouth-to-mouth ventilation" via pocket-face-mask and 95% for the LMA [11]. Recently Adelborg et al. proved "mouth-to-mouth" ventilation to reduce interruptions in chest compressions and producing a higher proportion of effective ventilation during lifeguard CPR, compared to "mouth-to-pocket-mask" or "bag-valve-mask" ventilation [25]. In consideration of these trials and acceptance of our results supraglottic airway devices might be an excellent or even superior alternative to established ventilation provided by lay responders.

We acknowledge that our study is subject to several limitations. Our results suggest that the majority of supraglottic airway devices are well suited to use by laypersons. This might imply in a preclinical emergency setting that laypersons, regardless of experience, can provide a secured airway and hence bridge the time until a professional resuscitation team is on site. On the other hand, it remains to be proven whether laypersons in a real resuscitation scenario, with its associated challenges, can still rely on their intuition. Secondly, it is beyond the scope of the present study to compare different educational modalities (classic, video, pictogram, practical or combined) with regard to their effectiveness [14,20]. Thirdly, skills when newly acquired and without subsequent practice are known to deteriorate [26], and it would therefore be worthwhile re-evaluating all participants in the near future. Recent findings however are promising: a single combined theoretical, video assisted and practical tutorial enables paramedical students to operate different supraglottic airway devices in a mannequin with retention of skills close to 100% after three months, even if no further clinical or manikin training is provided [18,27].

## Conclusion

In conclusion, we show that in a manikin resuscitation scenario, airway management can be safely and effectively performed by laypersons. The intuitive nature and ease of use of supraglottic airway devices clearly leads to high success rates and this is one of their key benefits. The present study shows that these benefits and success rates also extend to members of the public without formal healthcare training, and even without any knowledge of first-aid. Together with the positive attitude expressed by the participants towards such devices, our results recommend the incorporation of supraglottic airway devices in first aid and BLS courses.

## Disclosure

The authors declare that they have no competing interests.

## Authors' contributions

GS carried out conception and design, interpretation of data and drafted the manuscript. CS and SR participated in interpretation and helped to draft the manuscript. MC and MS critical revised the manuscript and supervised statistical analysis. MA allocated data and supervised setting and participants. RR approved the final version and NZ co-conceived, critical revised the manuscript and allocated data. All authors read and approved the final manuscript.
